# Mixed Fibronectin-Derived Peptides Conjugated to a Chitosan Matrix Effectively Promotes Biological Activities through Integrins, α4β1, α5β1, αvβ3, and Syndecan

**DOI:** 10.1089/biores.2016.0037

**Published:** 2016-11-01

**Authors:** Kentaro Hozumi, Kyotaro Nakamura, Haruna Hori, Mari Miyagi, Rika Nagao, Keiko Takasaki, Fumihiko Katagiri, Yamato Kikkawa, Motoyoshi Nomizu

**Affiliations:** Laboratory of Clinical Biochemistry, Department of Pharmacy, Tokyo University of Pharmacy and Life Sciences, Hachioji, Tokyo, Japan.

**Keywords:** artificial extracellular matrix, cell adhesive peptide, chitosan, fibronectin, integrin

## Abstract

Mimicking the biological function of the extracellular matrix is an approach to developing cell adhesive biomaterials. The RGD peptide, derived from fibronectin (Fn), mainly binds to integrin αvβ3 and has been widely used as a cell adhesive peptide on various biomaterials. However, cell adhesion to Fn is thought to be mediated by several integrin subtypes and syndecans. In this study, we synthesized an RGD-containing peptide (FIB1) and four integrin α4β1-binding-related motif-containing peptides (LDV, IDAPS, KLDAPT, and PRARI) and constructed peptide-chitosan matrices. The FIB1-chitosan matrix promoted human dermal fibroblast (HDF) attachment, and the C-terminal elongated PRARI (ePRARI-C)-conjugated chitosan matrix significantly promoted HDF attachment through integrin α4β1 and syndecan binding. Next, we constructed a mixed ePRARI-C- and FIB1-chitosan matrix to develop a Fn mimetic biomaterial. The mixed ePRARI-C/FIB1-chitosan matrix promoted significantly better cell attachment and neurite outgrowth compared to those of either ePRARI-C- or FIB1-chitosan matrices. HDF adhesion to the ePRARI-C/FIB1-chitosan matrix was mediated by integrin, α4β1, α5β1, and αvβ3, similar to HDF adhesion to Fn. These data suggest that an ePRARI-C/FIB1-chitosan matrix can be used as a tool to analyze the multiple functions of Fn and can serve as a Fn-mimetic biomaterial.

## Introduction

Fibronectin (Fn) is a large multidomain and multifunctional glycoprotein found in the extracellular matrix (ECM), on the cell surface, and in the extracellular fluid.^1^ Fn participates in cell adhesion, migration, ECM formation, thrombosis, and homeostasis.^2^ Fn contains two main cell adhesion domains located in the central, primary cell adhesive site and in the C-terminal, secondary cell adhesive site. It is believed that Fn promotes cell adhesion through both sites by various cell surface receptors.

ECM proteins generally contain many cell surface receptor-binding sites. A major ECM-binding group of cell-surface receptors is the integrin family. Many cell surface receptors that cross talk with integrins to regulate a multitude of cellular events have been identified.^3^ Thus, investigating cell–ECM interactions and mimicking the biological functions of the ECM using a single receptor targeting strategy are difficult due to the complexity of the mechanisms involved. RGD is the most well-known cell adhesive sequence and has been identified from the Fn primary cell adhesive site. RGD-containing peptides have been widely used to provide cell adhesive activity on various biomaterials.^4,5^ Although Fn promotes cell attachment through different cell surface receptors, generally RGD is used as the Fn-derived active peptide that promotes Fn's biological function.

Recently, we developed easy-handling peptide-chitosan matrices and examined their potential applications as biomaterials using *in vitro* and *in vivo* assays.^6,7^ For example, we conjugated the laminin α1 chain LG4 module active peptide, EF1zz (ATLQLQEGRLHFXFDLGKGR, X: Nle, mouse laminin α1 chain LG4 module 2749–2767 that binds to integrin α2β1), to a chitosan matrix. The EF1zz-chitosan matrix promotes integrin α2β1-mediated cell spreading with focal adhesion formation.^7^ We also demonstrated that an AG73 (RKRLQVQLSIRT, mouse laminin α1 chain LG4 module 2719–2730 that binds to syndecan)-chitosan matrix could deliver cells, such as keratinocytes, to the wound bed. We have also mixed different biologically active peptides and conjugated them to a polysaccharide matrix to develop a multifunctional artificial ECM. When we mixed EF1zz and AG73, the resulting AG73/EF1zz-chitosan matrix led to strong cell attachment, neurite outgrowth, and promoted the synergistic cooperation between integrin and syndecan receptors similar to the effects of recombinant laminin α1 chain LG4 protein.^8^ These findings demonstrate that the mixed peptide-chitosan matrix system is a powerful tool for constructing artificial ECM scaffolds that mimic the activity of intact ECM molecules and for investigating the molecular mechanisms among different cell surface receptors.

Here in, we focused on the multiactive ECM protein Fn. The active peptides from both the Fn-primary and the Fn-secondary cell adhesive sites were conjugated to the chitosan matrix, and the cell adhesive activities of the peptide-chitosan matrices were assessed. Then, the active peptides from the two different sites were mixed and the mixed peptide-chitosan matrix effectively promoted cell adhesion and neurite outgrowth that invoked synergistic cooperation of different receptors as Fn.

## Materials and Methods

### Antibodies

Specific antibodies directed against integrins α1 (FB12), α2 (P1E6), α3 (P1B5), α4 (P1H4), α5 (P1D6), α6 (GoH3), αv (AV1), β1 (6S6), and β3 (25E11) and a mouse polyclonal IgG (PP54) were purchased from AMAC (Westbrook, ME), Cell Signaling Technology (Beverly, MA), Santa Cruz Biotechnology (Santa Cruz, CA), and/or EMD Millipore (Billerica, MA).

### Synthetic peptides

All peptides were manually synthesized using the 9-fluorenylmethoxycarbonyl (Fmoc) strategy as previously described.^8^ For conjugation to a chitosan matrix, a cysteine residue was added at the N-terminus, and two glycine residues were used as a spacer between the cysteine and the active peptide sequence. The purity and identity of the peptides were confirmed by an electrospray ionization mass spectrometer.

### Cell culture

Human dermal fibroblasts (HDFs; Cell Applications, Inc., San Diego, CA) were maintained in Dulbecco's modified Eagle's medium (DMEM) containing 10% fetal bovine serum (FBS), 100 U/mL penicillin, and 100 mg/mL streptomycin (Pen/Strep; Invitrogen, Carlsbad, CA). The human lymphoid cell line ARH-77 was maintained in RPMI-1640 containing 5% FBS with Pen/Strep.^9^ Rat pheochromocytoma PC12 cells were cultured in DMEM/F-12 (Invitrogen) containing 7.5% horse serum (HS; Invitrogen) and 7.5% FBS with Pen/Strep.

### Cell attachment assay

The peptide-conjugated chitosan matrix was prepared in 96-well plates (Nalge Nunc, Inc., Rochester, NY) as described previously.^8^ Briefly, *N*-(*m*-maleimidobenzoyloxy) succinimide (MBS)-conjugated chitosan (MB-chitosan) was added to a 96-well plate (3 ng/mm^2^) and dried. Then, various concentrations of the peptides in a 0.1% TFA solution and a 1% NaHCO_3_ solution were added into the wells and incubated for 2 h. Then, the 96-well plates were blocked by the addition of 1% bovine serum albumin in DMEM for 1 h. Either HDFs or ARH-77 cells were added (100 μL, 2 × 10^4^ cells) to each well and incubated at 37°C for 60 or 90 min in 5% CO_2_. After washing off the unattached cells, the attached cells were stained with a 0.2% crystal violet aqueous solution in 20% methanol. The attached cells were photographed using a microscope and counted (Olympus, Tokyo, Japan).

For inhibition of cell attachment, HDFs were pretreated with 5 mM EDTA, 10 μg/mL heparin, or 10 μg/mL anti-integrin antibodies at room temperature for 20 min. Then, the cells were added to the wells (100 μL, 1 × 10^4^ cells/well) and incubated for 45 min at 37°C. The attached cells were counted as described above.

### Immunocytostaining

Immunocytostaining was carried out as previously described.^8^ The peptide-chitosan matrices were prepared onto an eight-well chamber slide (Nalge Nunc), and HDFs (300 μL, 5 × 10^3^ cells/well) were incubated at 37°C for 90 min. HDFs were fixed and incubated with anti-vinculin antibody (hVIN-1), then stained with Rhodamine red-labeled secondary antibody, Alexa Fluor 488-labeled phalloidin (Invitrogen), and DAPI. The glass slides were examined under a FluoView FV1000D IX81 microscope (Olympus).

### Neurite outgrowth assay

After priming with nerve growth factor (NGF 2.5S; 100 ng/mL) in DMEM/F-12 containing 30 nM of NaSeO_3_ (Wako) for 24 h, PC12 cells (100 μL, 5 × 10^3^ cells/well) were seeded into the peptide-chitosan matrix-coated 96-well plates (30 ng/mm^2^) in DMEM/F-12 containing 30 nM of NaSeO_3_, 100 ng/mL of NGF 2.5S, 100 mg/mL transferrin, 20 nM progesterone, and 5 mg/mL insulin. The cells were incubated for 24 h and then stained with 0.2% crystal violet.

## Results

### HDF-adhesion activity on various integrin-binding peptide-chitosan matrices

**FIG. 1.**
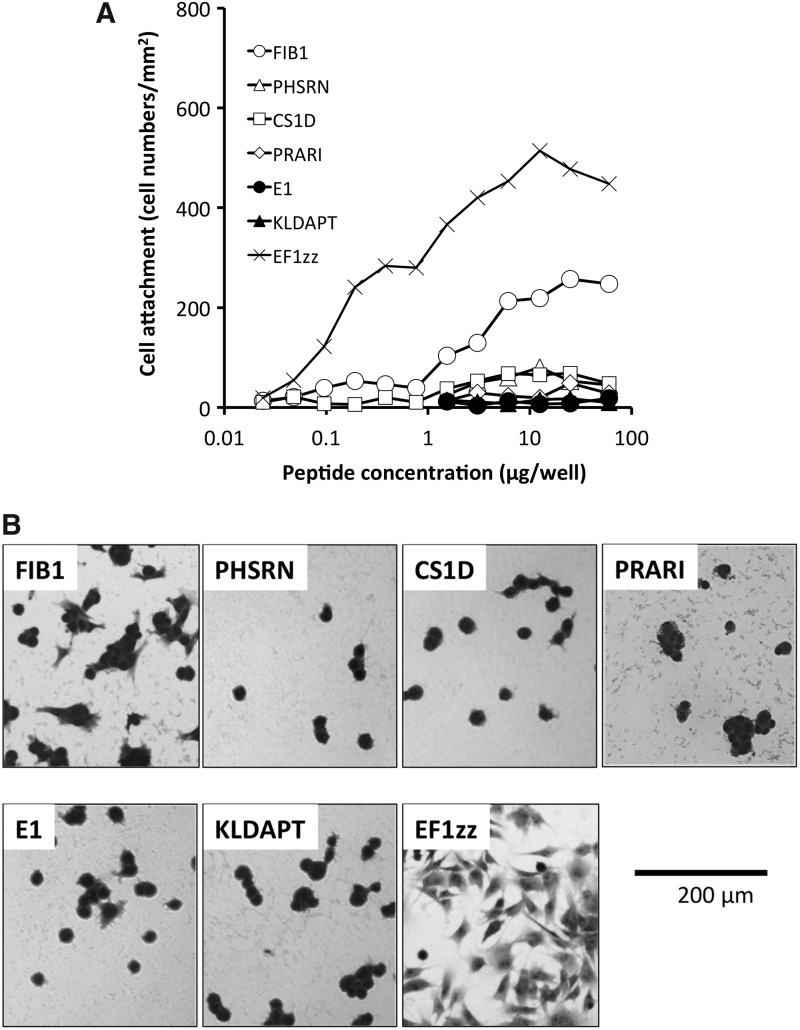
Fibroblast attachment activities to Fn-derived peptide-chitosan matrices. **(A)** Dose-dependent attachment activities and **(B)** morphological appearance of fibroblasts on the peptide-chitosan matrices. Six Fn-derived peptides CGG-FIB1 (integrin αvβ3 binding), CGG-PHSRN (integrin αvβ3-binding synergistic site), CGG-CS1D (integrin α4β1 binding), CGG-PRARI (integrin α4β1-binding synergistic site), CGG-E1 (integrin α4β1 binding), and CGG-KLDAPT (integrin α4β1 binding) were coupled to the MB-chitosan matrix in various concentrations in 96-well plates as described in the Materials and Methods section. A CGG-EF1zz (CGGATLQLQEGRLHFXFDLGKGR, X: Nle, mouse laminin α1 chain, integrin α2β1 binding)-chitosan matrix was used as control. Fibroblasts (2 × 10^4^ cells) were allowed to attach to the peptide-chitosan matrices for 90 min and then stained with crystal violet. The attached cells in the central fields of the well were counted using a microscope (mm^2^). Fibroblast morphology on 12.5 μg/well of each peptide-chitosan matrix was photographed. Triplicate experiments gave similar results, and data are expressed as mean ± SD of triplicate results. Scale bar indicates 200 μm. Fn, fibronectin.

We prepared seven cell adhesive peptides derived from either Fn or laminin that bind different integrin subtypes (Table 1). These integrin-binding peptides were conjugated to a MB-chitosan matrix and evaluated for their cell attachment activity using HDFs (Fig. 1A). EF1zz-chitosan matrix and FIB1-chitosan matrix showed strong HDF attachment activity in a dose-dependent manner. PHSRN- and CS1D-chitosan matrices weakly promoted HDF attachment, while PRARI-, KLDAPT-, and E1-chitosan matrices exhibited poor HDF attachment.

**Table 1.** **Peptide Used in Assays**

Peptide	Sequence	Domain	Receptors	References
FIB1	CGGYAVT*GRGDS*PAS	III10	Integrin αvβ3, α5β1	Mochizuki et al.^7^
PHSRN	CGG*PHSRN*	III9	RGD motif synergistic site	Aota et al.^11^
CS1D	CGGVTLPHPNLHGPEI*LDV*PST	IIICS	Integrin α4β1	Moyano et al.,^12^ Mould et al.^15^
PRARI	CGGP*PRARI*	III14	Integrin α4β1 binding synergistic site, syndecan	Sharma et al.,^17^ Woods et al.^18^
E1	CGGASTA*IDAPS*NLR	III14	Integrin α4β1	Mohri et al.^14^
KLDAPT	CGGQTT*KLDAPT*NLQ	III5	Integrin α4β1	Moyano et al.^12^
EF1zz	CGGATLQLQEGRLHFXFDLGKGR	Laminin α1	Integrin α2β1	Mochizuki et al.^7^

CGG residues were added to their N-terminus to conjugate on the MB-chitosan matrix (see the Materials and Methods section). Italics indicate the core integrin binding related residues in each peptide.

The morphology of the attached HDFs on the peptide-chitosan matrices differed depending on the peptide. FIB1- and EF1zz-chitosan matrices promoted a typical integrin-mediated spreading morphology (Fig. 1B). CS1D- and E1-chitosan matrices exhibited some spike-like cellular structures. The HDFs on the PHSRN- and KLDAPT-chitosan matrices showed a round cell morphology.

### The HDF attachment activity of elongated PRARI motif peptide-chitosan matrices

**FIG. 2.**
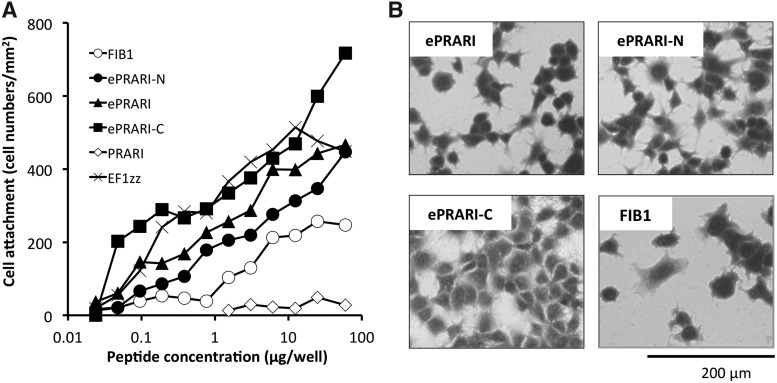
Fibroblast attachment activities to elongated PRARI motif peptide-chitosan matrices. **(A)** Dose-dependent attachment activities and **(B)** morphological appearance of fibroblasts on the peptide-chitosan matrices. Various concentrations of the three elongated PRARI motif peptides, CGG-ePRARI, CGG-ePRARI-N, and CGG-ePRARI-C, were conjugated to the MB-chitosan matrix in 96-well plates as described in the Materials and Methods section. FIB1- and EF1zz-chitosan matrices were used as control. Fibroblasts (2 × 10^4^ cells) were allowed to attach to the peptide-chitosan matrices for 90 min and then stained with crystal violet. The attached cells in the central fields of the well were counted while viewed in a microscope (mm^2^). The fibroblast morphology on 20 μg/well of each peptide-chitosan matrix was photographed. Triplicate experiments gave similar results, and data are expressed as mean ± SD of triplicate results. Scale bar indicates 200 μm.

Since four integrin α4β1-binding-related peptide-chitosan matrices had weak HDF attachment, we elongated the amino acid sequence of the PRARI motif, which was the shortest peptide tested, toward the N-terminus, C-terminus, and both termini (Table 2). Three different elongated PRARI-motif peptides were synthesized; each was 12 amino acids with CGG residues on its N-terminus. Amino acid addition onto the N-terminus was named ePRARI-N, addition to the C-terminus was named ePRARI-C, and ePRARI was coined for elongation of both termini. HDF attachment activities of the three elongated PRARI peptide-conjugated chitosan matrices were evaluated (Fig. 2A). The three elongated PRARI-chitosan matrices exhibited strong HDF attachment, respectively. The ePRARI-C-chitosan matrix promoted significantly stronger HDF attachment activity compared with the EF1zz-chitosan matrix. The HDFs on the three elongated PRARI-chitosan matrices exhibited a spread and elongated morphology, indicating that HDF attachment on the three elongated PRARI-chitosan matrices involves integrin-mediated HDF attachment (Fig. 2B).

**Table 2.** **Amino Acid Sequences and Their Location in Fibronectin of Elongated PRARI Peptides**

Peptide	Sequence	Location of hFn (aa)
PRARI	CGGP*PRARI*	2016–2021
ePRARI-N	CGGLVSWQP*PRARI*T	2011–2023
ePRARI	CGGSWQP*PRARI*TGY	2013–2025
ePRARI-C	CGGQP*PRARI*TGYII	2015–2027

CGG residues were added to their N-terminus to conjugate on the MB-chitosan matrix (see the Materials and Methods section). Italics indicate the core PRARI residue in each peptide.

Fn, fibronectin.

### Inhibitory effect of anti-integrin antibodies on HDF adhesion to the peptide-chitosan matrices

**FIG. 3.**
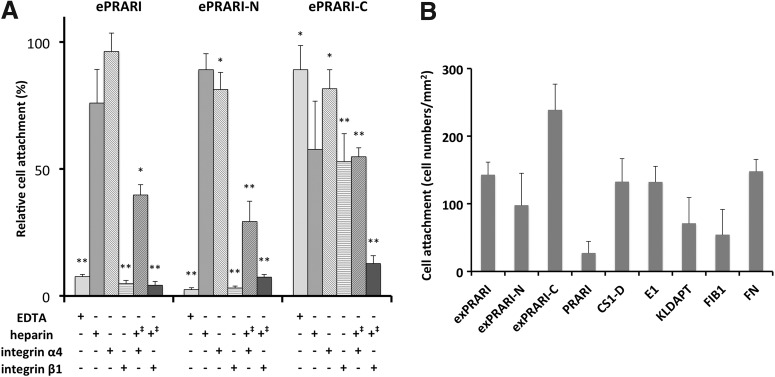
Inhibitory effect of heparin, EDTA, and anti-integrin function blocking antibodies on fibroblast attachment to elongated PRARI peptide-chitosan matrices **(A)** and lymphoid cell (ARH-77) attachment activities to Fn-derived peptide-chitosan matrices **(B)**. **(A)** Three elongated PRARI peptides were coupled to the MB-chitosan matrix in 96-well plates as described in the Materials and Methods section. Fibroblasts (1 × 10^4^ cells) were preincubated with either 5 mM EDTA, 10 μg/mL of heparin, or specific antibodies directed against either integrin α4 (P1H4) or β1 (6S6) and a negative control of mouse purified polyclonal IgG (PP54). For double inhibition by heparin and anti-integrin antibodies, the concentration of heparin was reduced to 0.001 μg/mL (+‡) that would not affect HDF attachment. After a 20 min incubation, fibroblasts were allowed to attach to the peptide-chitosan matrices for 45 min and then stained with crystal violet. Relative cell attachment activities were calculated against the negative control of mouse IgG, and data are expressed as mean ± SD of triplicate results. **p < 0.01, *p < 0.1 against cell attachment activity of HDF treated by polyclonal IgG. **(B)** Four Fn-derived integrin α4β1-binding peptides, CS1D, PRARI, E1, and KLDAPT, and three elongated PRARI peptides (10 μg/well) were coupled to the MB-chitosan matrix in 96-well plates as described in the Materials and Methods section. Fn (2 μg/well) was coated on the plate by a 2 h incubation at room temperature and served as a control. ARH-77 cells (2 × 10^4^ cells/well) were allowed to attach to the peptide-chitosan matrices for 60 min and then stained with crystal violet. The attached cells in the central fields of the wells were counted. Triplicate experiments gave similar results, and data are expressed as mean ± SD of triplicate results.

Next we assessed whether HDF attachment to the three elongated PRARI-chitosan matrices was mediated by integrin α4β1 using anti-integrin α4 or β1 function-blocking antibodies, EDTA, or heparin pretreated HDFs. HDF attachment to the ePRARI- and ePRARI-N-chitosan matrices was significantly inhibited by EDTA and anti-integrin β1 antibody and moderately inhibited by heparin (Fig. 3). These findings suggested that HDF attachment to these two peptide-chitosan matrices was mainly promoted by integrin β1-mediated cell adhesion and partially by heparin-mediated cell adhesion, such as using syndecans. Syndecans are members of a family of cell surface proteoglycans, and cell-adhesion through syndecans is inhibited by heparin.^8^ HDF attachment to the ePRARI-C-chitosan matrix was inhibited by heparin and by anti-integrin β1 antibody and moderately inhibited by EDTA and by anti-integrin α4 antibody. Since HDF attachment to the three elongated PRARI-chitosan matrices was not clearly inhibited by both anti-integrin α4 or β1 antibodies, we next pretreated HDFs with a mixture of anti-integrin antibodies and a low concentration of 0.001 μg/mL heparin that would not affect HDF attachment to the three peptide-chitosan matrices (data not shown). HDF attachment to the three elongated PRARI-chitosan matrices was inhibited by both integrin α4 and β1 function-blocking antibodies with heparin, suggesting that HDF attachment was mediated by integrin α4β1 and by syndecans.

### ARH-77 cell attachment activity to the elongated PRARI motif peptide-chitosan matrix

**FIG. 4.**
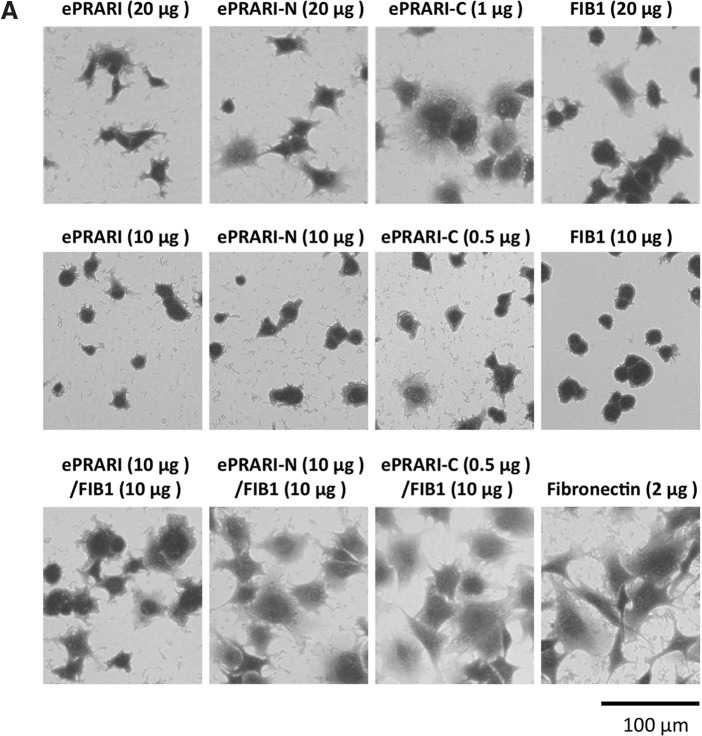

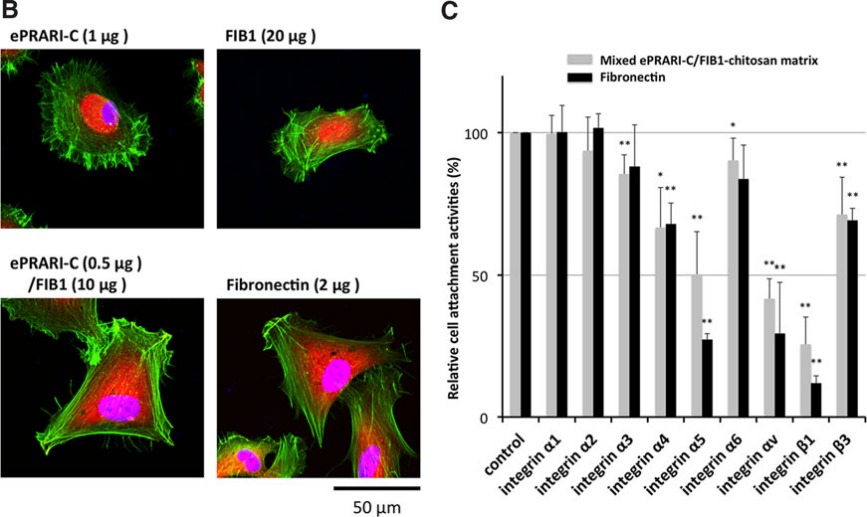
Fibroblast attachment and spreading activity **(A)** and actin cytoskeleton and focal adhesion organization **(B)** on mixed peptide-conjugated chitosan matrices. FIB1 from primary cell adhesion site and three elongated PRARI peptides from the secondary cell adhesion site were used to examine the synergistic cross talk between different cell surface receptors. FIB1 (20 μg/well), ePRARI (20 μg/well), ePRARI-N (20 μg/well), ePRARI-C (1 μg/well), and the mixture of FIB1/ePRARI (10/10 μg/well), FIB1/ePRARI-N (10/10 μg/well), and FIB1/ePRARI-C (10/0.5 μg/well) were coupled to the MB-chitosan matrices. Fn (2 μg/mL) was served as a control. The fibroblasts (2 × 10^4^ cells/well) were allowed to attach to the peptide-conjugated chitosan matrices for 60 min and then stained with crystal violet **(A)**. Immunocytostaining has been done as actin cytoskeleton (green), vinculin (red), and nuclei (blue). Scale bar indicates 100 μm **(A)** and 50 μm **(B)**. Triplicate experiments gave similar results. **(C)** Inhibitory effect of various anti-integrin function blocking antibodies on HDF attachment to either a mixed FIB1/ePRARI-C-chitosan matrix or Fn. FIB1/ePRARI-C (10/0.5 μg/well) was coupled to the MB-chitosan matrix. Fibroblasts (1 × 10^4^ cells) were preincubated with 10 μg/mL of specific antibodies directed against integrins, α1, α2, α3, α4, α5, α6, αv, β1, and β3, and a negative control of mouse purified polyclonal IgG. After a 20 min incubation, fibroblasts were allowed to attach to the peptide-chitosan matrices or Fn for 60 min and then stained with crystal violet. Relative cell attachment was calculated against the negative control of mouse IgG, and data are expressed as mean ± SD of triplicate results. **p < 0.01, *p < 0.1 against cell attachment activity of HDF treated by polyclonal IgG.

ARH-77 lymphoid cells were established from a patient with plasma cell leukemia and express integrin α4β1^10^ extensively and low levels of syndecans.^9^ Therefore, the cell adhesion to peptide-chitosan matrices was investigated using ARH-77 cells (Fig. 4). CS1D-, E1-, KLDAPT-, ePRARI-, and ePRARI-N-chitosan matrices promoted ARH-77 attachment similar to that of Fn (2 μg/well), but the PRARI-chitosan matrix was inactive. The ePRARI-C-chitosan matrix promoted the strongest cell attachment activity. FIB-conjugated chitosan matrix weakly promoted ARH-77 attachment. This is expected as ARH-77 cells express the RGD-binding integrin subtype, integrin αvβ3, and a lower level (90% less) of integrin α4β1.^10^ These findings suggested that ePRARI- and ePRARI-N-chitosan matrices promoted integrin α4β1-mediated cell attachment in the same manner as that of other traditional integrin α4β1-binding peptide-chitosan matrices. ARH-77 attachment to the ePRARI-C-chitosan matrix was dramatically increased indicating that ePRARI-C-chitosan matrix effectively promoted integrin α4β1 binding mediated cell adhesion.

### Mixed peptide-chitosan matrix effectively promotes biological activities through cross talk of different receptors

To mimic the cross talk involved in Fn activity, we mixed FIB1 with the three elongated PRARI peptides and prepared mixed peptide-chitosan matrices. HDF attachment to FIB1-, ePRARI-, and ePRARI-N-peptide chitosan matrices reached a plateau with almost 300–400 cells/mm^2^ when 20 μg/well of each peptide was reacted with the MB-chitosan matrix, and the ePRARI-C-peptide chitosan matrix reached 400 cells/mm^2^ at 1 μg/well (Fig. 1A). The four peptide-chitosan matrices promoted almost the same level of HDF attachment with the cells showing an elongated morphology that is typically found with integrin-mediated adhesion (Fig. 4A, upper row). The ePRARI-C-chitosan matrix promoted significantly more HDF spreading than the other three peptide-chitosan matrices. Next, we mixed half of the amount of each peptide and conjugated them to the MB-chitosan matrix to evaluate HDF attachment. The three mixed peptide-chitosan matrices effectively promoted HDF attachment and HDF spreading more compared with the single peptide-chitosan matrices (Fig. 4A, lower row). Specifically, the ePRARI-C/FIB1-chitosan matrix promoted HDF attachment significantly, and the cellular morphology and the organized actin cytoskeleton were similar to the Fn-coated plates (Fig. 4B).

We evaluated the inhibitory effect of various anti-integrin function-blocking antibodies on HDF attachment to the mixed ePRARI-C/FIB1-chitosan matrix and to Fn. HDF attachment on the mixed ePRARI-C/FIB1-chitosan matrix was inhibited by anti-integrin α5, αv, and β1 antibodies (Fig. 4C). Less inhibition was seen with anti-integrin α4 and β3 antibodies. HDF attachment to Fn was significantly inhibited by anti-integrin α5, αv, and β1 antibodies and relatively less inhibited by anti-integrin α4 and β3 antibodies. These results suggested that both the mixed ePRARI-C/FIB1-chitosan matrix and Fn promoted HDF attachment mainly through the integrins α4β1, α5β1, and αvβ3.

**FIG. 5.**
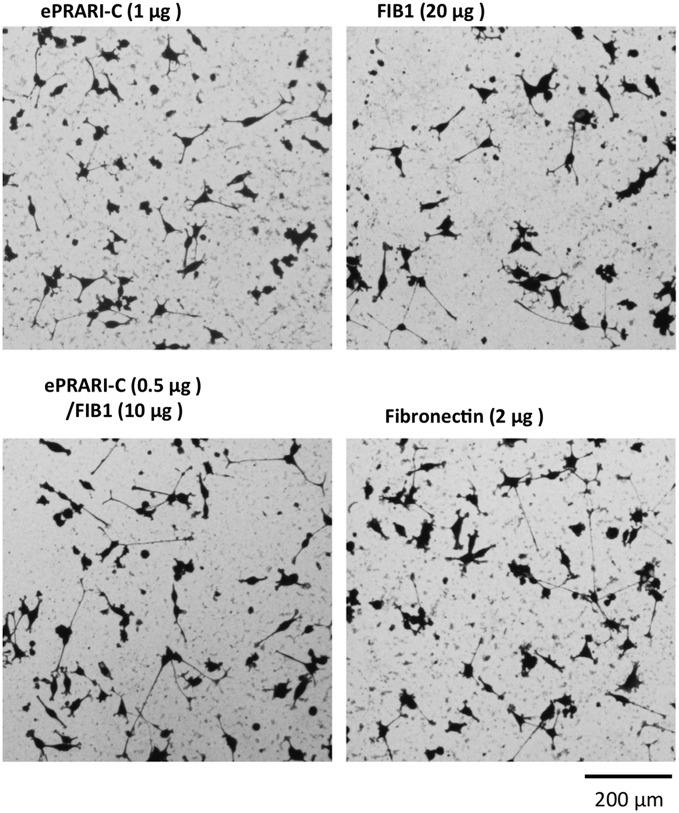
PC12 cell neurite outgrowth activities on ePRARI-C/FIB1-chitosan matrix. FIB1 (20 μg/well), ePRARI-C (1 μg/well), ePRARI-C/FIB1 (10/0.5 μg/well) were coupled to the MB-chitosan matrices as described in the Materials and Methods section. Fn (2 μg/mL) was served as a control. The primed PC12 cells (5 × 10^3^ cells/well) were allowed to attach to the peptide-conjugated chitosan matrices or Fn with several supplements to promote the neurite outgrowth for 24 h and then stained with crystal violet. Scale bar indicates 200 μm. Triplicate experiments gave similar results.

We also tested cell neurite outgrowth activities of PC12 cells and SK-N-SH cells with the ePRARI-C/FIB1-chitosan matrix and found that the ePRARI-C/FIB1-chitosan matrix effectively promoted neurite outgrowth similar to Fn-coated plates (Fig. 5 and Supplementary Fig. S1). Hence, a mixture of 0.5 μg of ePRARI-C and 10 μg of FIB1 conjugated to a chitosan matrix effectively promoted HDF attachment, and its biological activity was similar to that of a plate coated with 2 μg/well of Fn alone.

## Discussion

In this study, we mixed FIB1 peptide, containing the RGD motif, and the Fn-derived integrin α4β1-binding peptide of ePRARI-C to construct an ePRARI-C/FIB1-chitosan matrix. The ePRARI-C/FIB1-chitosan matrix significantly promoted cell adhesion, spreading, and neurite outgrowth compared to cells grown on Fn. However, less than half of the amount of peptide conjugated to the chitosan matrix was needed compared to FIB1 alone. We previously found that FIB1-chitosan matrix promoted HDF attachment mediated by integrin subtype-specific binding in a dose-dependent manner.^7^ PHSRN-chitosan, a synergistic integrin α5β1-binding site of RGD,^11^ weakly promoted HDF attachment. Four well-known integrin α4β1-binding sequences of CS1D, E1, PRARI, and KLDAPT were selected for development of an integrin α4β1-binding peptide-chitosan matrix (Table 1). CS1D, E1, and PRARI are derived from the 29-kDa fragment of the secondary cell-adhesion site of Fn, and KLDAPT was identified from the III5 domain by alignment with CS-1 and used as a control.^12^ While the CS1D-chitosan matrix weakly promoted HDF attachment, the other three peptide-chitosan matrices showed little activity. In contrast, ARH-77 cell attachment was enhanced on all integrin α4β1-binding-related peptide-chitosan matrices. The cell attachment activity of the elongated ePRARI-C-chitosan matrix and ePRARI-alginate matrix (unpublished data) was 10-fold stronger compared with the nonelongated PRARI. Fibroblast expression of integrin α4β1 is low relative to integrin α5β1 expression.^13^ In most cases, these integrin α4β1-binding-related peptides inhibited integrin α4β1-mediated cell attachment and spreading when they were either conjugated to globular proteins such as KLH or added as soluble peptides.^12,14,15^ These findings suggest the possibility that peptide conjugation to chitosan may allow the peptide to adopt a conformation that is different from when it is conjugated to globular proteins.

The PRARI motif was identified from the major active site of peptide FN-C/H-V (WQPPRARI; human Fn, aa 1892–1899) on 29-kDa fragment of Fn. FN-C/H-V mediates cell adhesion through a cell surface proteoglycan and promotes focal adhesion formation. The heparin-binding activity of the 29-kDa fragment was significantly inhibited by the PRARI-containing peptide, and integrin-mediated focal adhesions formed by trabecular meshwork cells were also disrupted by PRARI peptide treatment.^16^ The crystal structural analysis using mutant 29-kDa fragment revealed that two Arg residues in the PRARI motif are important for both heparin and integrin α4β1 binding.^17^ Thus, the PRARI motif was proposed as the synergistic integrin α4β1-binding site in the 29-kDa fragment based on crystal structure analysis.^13^ Three ePRARI motif peptides conjugated to chitosan matrices significantly promoted fibroblast attachment in agreement with previous results.

With the exception of PPRARI, integrin α4β1-binding related peptides are longer (12–18 residues) than the 12 residue RGD-containing FIB1 peptide. To enhance HDF attachment to the integrin α4β1-binding-related peptides, we focused on the shortest peptide of PRARI and elongated it at the N and/or the C terminus to 12 residues. Surprisingly, three elongated PRARI peptide-conjugated chitosan matrices significantly promoted HDF attachment by 20-fold or more compared to the PRARI-chitosan matrix. Among the elongated PRARI-chitosan matrices, the C-terminal elongated ePRARI-C-chitosan matrix promoted the strongest HDF attachment. It has been reported that several different lengths of PRARI-containing FN-C/H-V have been described. The shortest FN-C/H-V is eight residues (WQPPRARI), and the longest is 18 residues (QPPRARITGYIIKYEKPG).^18–20^ Originally, FN-C/H-V was identified as the eight amino acid sequence, but many reports elongated the sequences on the C-terminal side.^19,20^ These findings support the hypothesis that C-terminal extension of the PRARI motif strengthens the cell attachment activity of PRARI. We also elongated the second shortest peptide of PHSRN to 12 residues. However, HDF attachment to the elongated PHSRN-chitosan matrix showed no change (data not shown). Furthermore, HDF attachment to the mixed elongated PHSRN/FIB1-chitosan matrix was the same as that of the FIB1-chitosan matrix.

Many studies have shown that cross talk between integrins and other cell surface receptors accelerated cellular activities. For example, vitronectin interacts with both syndecan-1 and integrins and regulates cell spreading, as well as the assembly of focal contacts.^21,22^ We showed that the laminin α1 chain LG4 module efficiently promoted HDF attachment, spreading, and PC12 neurite outgrowth through the synergistic cross talk of integrin α2β1 and syndecans. The cross talk between syndecans and integrins by cells on Fn has been well defined in cell migration. Fn binds to integrins α4β1, α5β1, and αvβ3 and to syndecans.^23^ Binding to syndecan-2 and syndecan-4 induced cell adhesion and focal contact formation through integrin α5β1.^3^ Cell surface chondroitin sulfate binding promotes cell adhesion and focal contact formation through activated integrin α4β1, but the activation of integrin α4β1 is independent of syndecan-4 binding.^24^ Faster integrin α4β1 binding promotes subsequent integrin αvβ3 binding, and integrin α4β1 binding induces recognition of the RGDS sequence.^25^ Taken together, the mixed ePRARI-C/FIB1-chitosan matrix promotes fibroblast adhesion and spreading through cross talk by integrins, α4β1, α5β1, and αvβ3, and syndecans, and this combination mimics the cellular response in a manner similar to that of Fn.

## Supplementary Material

Supplemental data

## Abbreviations Used

ECM: extracellular matrix
DMEM: Dulbecco's modified Eagle's medium
FBS: fetal bovine serum
Fn: fibronectin
HDF: human dermal fibroblast
NGF: nerve growth factor
Pen/Strep: penicillin/streptomycin
