# Supplementary material for: Mixed Fibronectin-Derived Peptides Conjugated to a Chitosan Matrix Effectively Promotes Biological Activities through Integrins, α4β1, α5β1, αvβ3, and Syndecan
Source: Biores Open Access. 2016 Nov 1;5(1):356–66. doi: 10.1089/biores.2016.0037 (PMC5144869; doi:10.1089/biores.2016.0037)
Supplement: Supplemental data [file Supp_Fig1.pdf]

Supplementary Data

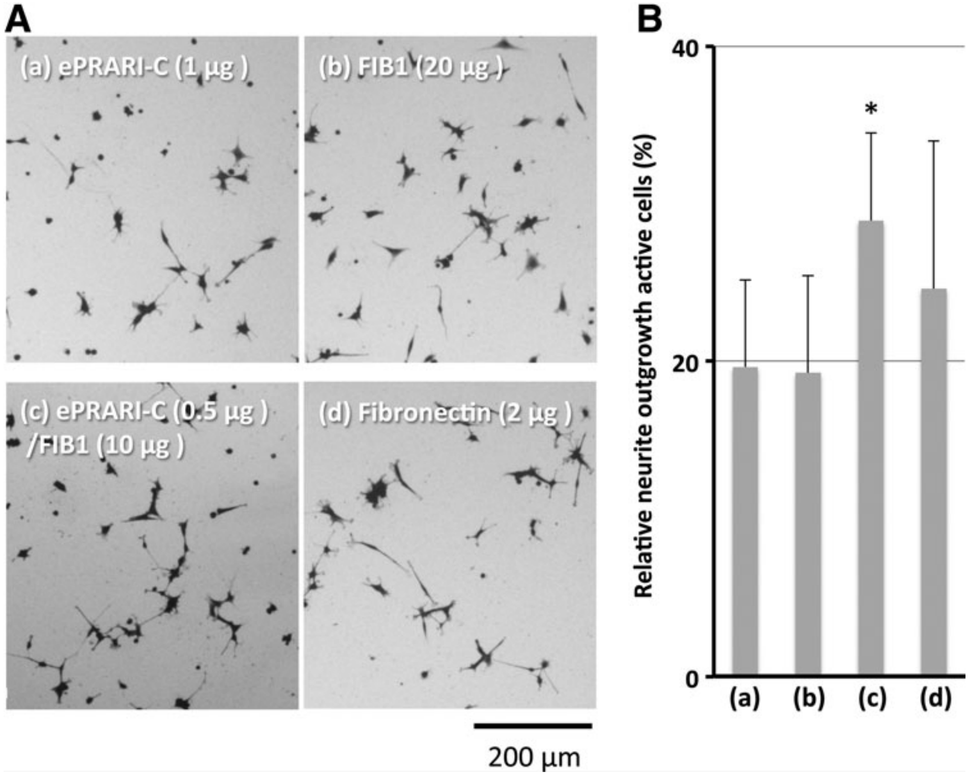

SUPPLEMENTARY FIG. S1. Human neuroblastoma cell line (SK-N-SH cells; this cell line was provided by the Riken BRC through the National Bio-Resource Project of the MEXT, Japan) were maintained in MEM-α containing 10% FBS, 100 U/ml penicillin, and 100 mg/ml. After priming with nerve growth factor (NGF 2.5S; 100 ng/ml) in MEM-α for 24 h, SK-N-SH cells (100 μl, 5×10^3 cells/well) were seeded into the peptide-chitosan matrix-coated 96-well plates (MB-chitosan; 30 ng/mm^2) in MEM-α with ITS-G supplement (Invitrogen). The cells were incubated for 24 h, then stained with 0.2% crystal violet. Neurite outgrowth of SK-N-SH cells on (a) ePRARI-C-chitosan matrix, (b) FIB1-chitosan matrix, (c) ePRARI-C/FIB1-chitosan matrix, and (d) Fibronectin were photographed; **(A)** The neurite outgrowth active cells were counted (if the length of neurites are double against cell body, the cells are counted as positive cells); **(B)** Triplicate experiments gave similar results, and data are expressed as mean ± SD of triplicate results. Scale bar indicates 200 μm. *$p < 0.01$.
